# Insight into the Web of Stress Responses Triggered at Gene Expression Level by Porphyrin-PDT in HT29 Human Colon Carcinoma Cells

**DOI:** 10.3390/pharmaceutics13071032

**Published:** 2021-07-07

**Authors:** Maria Dobre, Rica Boscencu, Ionela Victoria Neagoe, Mihaela Surcel, Elena Milanesi, Gina Manda

**Affiliations:** 1 Radiobiology Department, Victor Babes National Institute of Pathology, 99-101 Splaiul Independentei, 050096 Bucharest, Romania; maria_dobre70@yahoo.com (M.D.); neagoevictoria@gmail.com (I.V.N.); msurcel2002@yahoo.com (M.S.); elena.k.milanesi@gmail.com (E.M.); 2 Faculty of Pharmacy, Carol Davila University of Medicine and Pharmacy, 6 Traian Vuia Street, 020956 Bucharest, Romania; rboscencu@yahoo.com

**Keywords:** photodynamic therapy, colon carcinoma cells, stress responses

## Abstract

Photodynamic therapy (PDT), a highly targeted therapy with acceptable side effects, has emerged as a promising therapeutic option in oncologic pathology. One of the issues that needs to be addressed is related to the complex network of cellular responses developed by tumor cells in response to PDT. In this context, this study aims to characterize in vitro the stressors and the corresponding cellular responses triggered by PDT in the human colon carcinoma HT29 cell line, using a new asymmetric porphyrin derivative (P2.2) as a photosensitizer. Besides investigating the ability of P2.2-PDT to reduce the number of viable tumor cells at various P2.2 concentrations and fluences of the activating light, we assessed, using qRT-PCR, the expression levels of 84 genes critically involved in the stress response of PDT-treated cells. Results showed a fluence-dependent decrease of viable tumor cells at 24 h post-PDT, with few cells that seem to escape from PDT. We highlighted following P2.2-PDT the concomitant activation of particular cellular responses to oxidative stress, hypoxia, DNA damage and unfolded protein responses and inflammation. A web of inter-connected stressors was induced by P2.2-PDT, which underlies cell death but also elicits protective mechanisms that may delay tumor cell death or even defend these cells against the deleterious effects of PDT.

## 1. Introduction

Photodynamic therapy (PDT) has lately emerged as a promising targeted therapy for solid tumors. As reviewed by Agostinis et al. [1], PDT consists of the administration of a biocompatible photosensitizer (PS) that is inactive in “dark” conditions and is more or less selectively accumulated by tumor cells. Local activation of PS using visible light in a PS-specific wavelength spectrum triggers a strong singlet oxygen burst that induces locally important oxidative damages and therefore destabilizes the tumor niche. Because PSs should not exhibit “dark” cytotoxicity, and the activating light is highly targeted to the diseased tissue, PDT has minimal effects on healthy tissues. PDT has fewer side-effects than conventional anticancer therapies like radio- and chemotherapy and is sufficiently safe for repeated therapy sessions. Moreover, PDT does not induce immune suppression and can even boost the antitumor immune response which will complement the therapeutic action [2]. Major technical issues raised by PDT, recently summarized [3], are related to PS properties which, besides having to be non-cytotoxic in “dark” conditions, should have convenient amphiphilic properties to load cells and minimal aggregation in physiologic fluids and be activated with light of sufficiently high wavelength to penetrate tissues. Additionally, technical limitations in PDT are mostly related to the devices through which light can be guided to deep-seated tumors for activating PDT locally. An interesting issue for further improving PDT is the characterization of the complex mechanisms underlying its therapeutic effects and the potential therapy-induced resistance [4], both aspects being highly dependent on the tumor type, the structure and properties of the PS and on the PDT settings in terms of light wavelength and fluence. Particular aspects characterizing PDT-induced responses in tumor cells have been already documented, connecting the PDT-induced oxidative stress, cell death, survival mechanisms and danger-associated molecular patterns (DAMPs) production and immunity [5,6,7], but a detailed picture of the intra-network connections is still missing.

**Figure 1:**
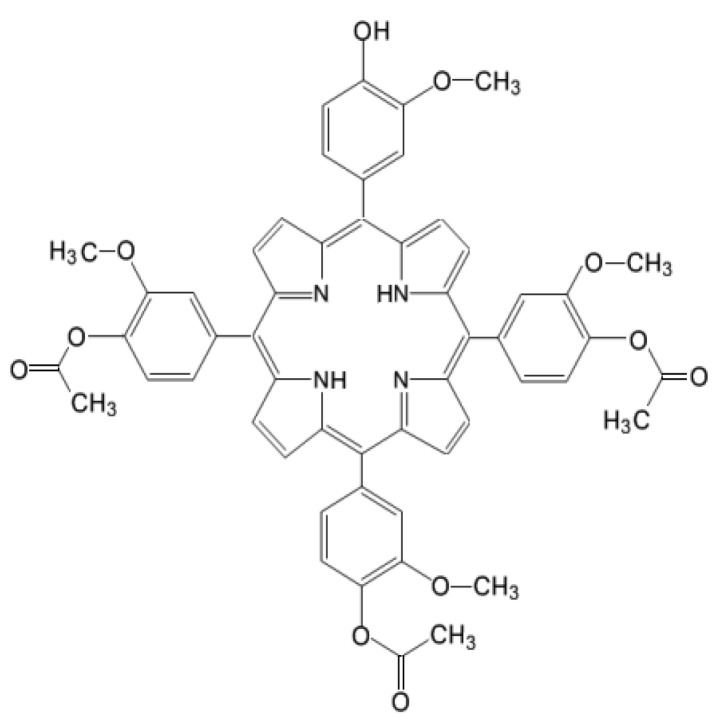
Chemical structure of 5-(4-hydroxy-3-methoxyphenyl)-10, 15, 20-tris-(4-acetoxy-3-methoxyphenyl) porphyrin (P2.2).

For characterizing at the molecular level the response of tumor cells to PDT, we investigated by qRT-PCR the gene expression pattern in HT29 tumor cells subjected to PDT, addressing 84 genes that are critically involved in cellular responses to stress, aiming to describe the web of stressors elicited by a porphyrin-PDT regimen. As a porphyrinic photosensitizer we used an unsymmetrical meso-tetrasubstituted phenyl porphyrin (4-acetoxy-3-methoxyphenyl)porphyrin, P2.2) that we have previously designed and characterized (Figure 1). As described in [8], P2.2 has remarkable amphyphylic properties, has good solubility in biologically friendly media and long-term stability in polyethylen glycol 200 (PEG 200) and is able to generate PDT-acceptable singlet oxygen yields when activated with light in the spectral domain 600–650 nm. P2.2 was shown to accumulate well into tumor cells following a dose-dependent linear relationship, was significantly less uptaken by blood cells, exhibited good fluorescence for imagistic detection and did not exert in “dark” conditions important in vitro cytotoxicity on cells specific for the tumor niche (tumor colon carcinoma cells and tumorigenic fibroblasts) or on blood cells (peripheral blood mononuclear cells). 

Results showed that the new P2.2 photosensitizer produced in vitro PDT in a concentration- and fluence-dependent manner. The gene expression study highlighted that P2.2-PDT generated a particular molecular fingerprint of oxidative stress, hypoxia signaling, DNA damage, endoplasmic reticulum (ER) stress and unfolded protein response (UPR), along with inflammation. The web of inter-connected stress responses elicited by P2.2-PDT in HT29 tumor cells might sustain or limit the therapeutic effect of PDT. The identified genes could represent valuable molecular targets for co-therapies aimed at reinforcing PDT.

## 2. Results

In vitro PDT was performed on the human colon carcinoma cell line HT29 using the new porphyrinic photosensitizer P2.2 (Figure 1) and activating laser light of 635 nm. 

**Figure 2:**
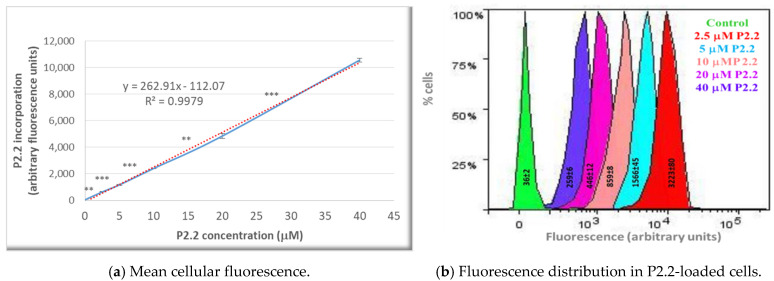
The uptake of the fluorescent P2.2 photosensitizer by HT29 tumor cells. Cells were incubated for 24 h with 2.5–40 µM P2.2. Intracellular P2.2 was evaluated by flow cytometry, based on the red P2.2 fluorescence. Fluorescence data, expressed in arbitrary units, and their median values in each sample were obtained with the BD FACSDiva software (Becton Dickinson). (**a**) Median cellular fluorescence. Data are presented as the mean value of the median fluorescence of each sample of the triplicate (mean ± SEM). Comparison between samples treated with consecutive P2.2 concentrations was performed using Student’s *t*-test considering unequal variances: ** *p* < 0.01, *** *p* < 0.001. (**b**) Fluorescence distribution in samples treated with various P2.2 concentrations. The represented SD values, obtained with the BD FACSDiva 6.1 software, are a measure of the fluorescence values spread around the mean value in a defined cellular population.

The fluorescent P2.2 photosensitizer was incorporated into HT29 tumor cells following a linear concentration-dependent relationship in the range (2.5–40) µM (Figure 2a). The difference between fluorescence values in samples loaded with consecutive P2.2 concentrations was statistically significant (*p* < 0.01). In the investigated concentration range, P2.2 did not exert “dark” cytotoxicity, according to the lactate dehydrogenase (LDH) release data (not shown). Additionally, the distribution of fluorescence values within a P2.2-treated sample and the corresponding SD value of the distribution (measure of the fluorescence values spread around the mean value in a defined cellular population) showed that cells had incorporated variable amounts of P2.2 (Figure 2b). This may affect the strength of PDT, depending on the cellular P2.2 load. 

The viability of tumor cells following exposure to P2.2-PDT was investigated as a preparative step for the gene expression analysis in PDT-treated samples as compared to non-treated controls.

### 2.1. PDT-Induced Changes of Cell Viability

#### 2.1.1. PDT-Induced Decrease of Viable Tumor Cells 

**Figure 3:**
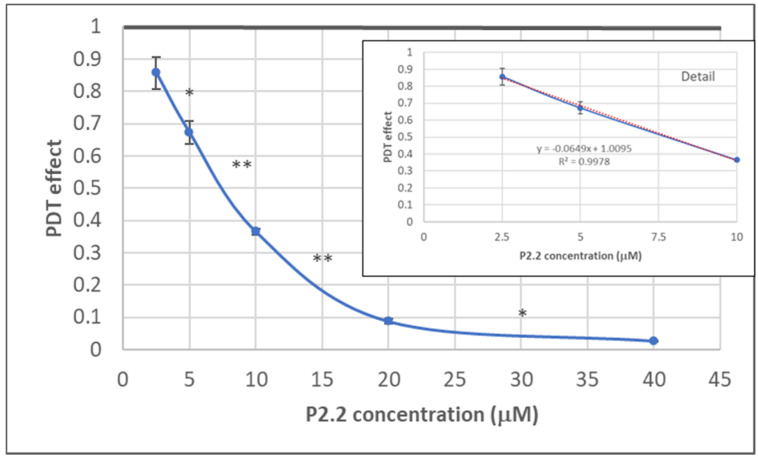
The dependence on P2.2 concentration of MTS reduction by HT29 tumor cells at 24 h post-PDT. Results are presented as PDT effect (mean ± SEM) in triplicate samples. PDT effect was calculated as OD in PDT-treated samples divided by the mean value of OD in control samples. Comparison between samples was performed using Student’s *t*-test: paired two sample for means: * *p* < 0.05, ** *p* < 0.01.

The dependence of the in vitro PDT outcome (viability of HT29 tumor cells) on P2.2 concentration (2.5–40 µM) was investigated by MTS ([3-(4,5-dimethylthiazol-2-yl)-5-(3-carboxymethoxyphenyl)-2-(4-sulfophenyl)-2H-tetrazolium, inner salt]) reduction at 24 h post-PDT (10 J/cm^2^, 50 mW/cm^2^). As shown in Figure 3, MTS reduction had a linear decrease in the P2.2 concentration range 2.5–10 µM. In the case of higher P2.2 concentrations (20–40 µM), MTS reduction almost dropped to zero, indicating that only few metabolically active cells remained at 24 h after PDT with high P2.2 concentrations.

An IC50 value of 8.07 ± 1.69 µM (mean ± SD) was computed using the Quest Graph™ IC50 Calculator (https://www.aatbio.com/tools/ic50-calculator, accessed on 27 June 2021). A concentration of 10 µM P2.2 was further chosen for performing PDT at various light fluences (5–25 J/cm^2^), for further differential investigation of both live and dead cells regarding gene expression.

**Figure 4:**
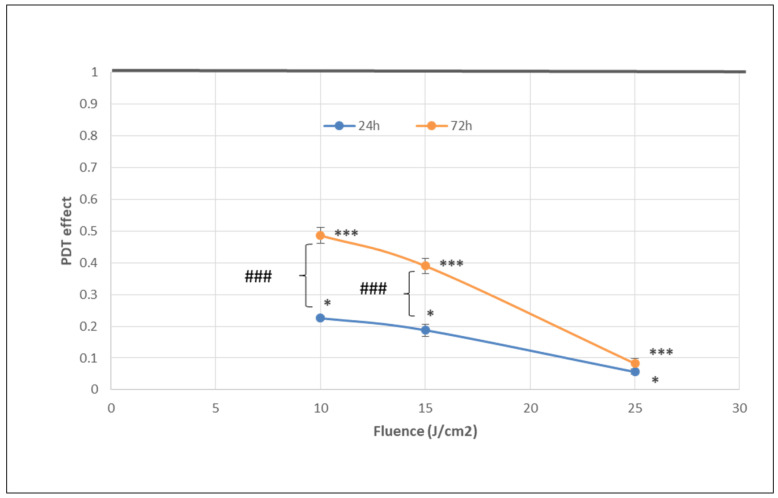
The dependence of MTS reduction by PDT-treated HT29 tumor cells on the fluence of the activation light delivered at a fluence rate of 50 mW/cm^2^. Results are presented as PDT effect (mean ± SEM) in triplicate samples. PDT effect was calculated as OD in PDT-treated samples divided by the mean value of OD in control samples. Comparison between samples was performed using Student’s *t*-test considering unequal variances: comparison between PDT regimens at a specific time-point: * *p* < 0.05, *** *p* < 0.001; comparison between samples at 24 h and 72 h: ### *p* < 0.001.

The number of metabolically active tumor cells in culture dramatically decreased at 24 h post-PDT in comparison with non-treated control cells, as shown by the significant fluence-dependent decrease of MTS reduction (PDT effect < 0.3) at all the tested light fluences (10 J/cm^2^, 15 J/cm^2^ and 25 J/cm^2^, delivered at 50 mW/cm^2^) (Figure 4). A subunit PDT effect (≤0.5) following a fluence-dependent curve was also registered at 72 h (Figure 4). A stronger PDT regimen (25 J/cm^2^) rendered almost all tumor cells metabolically inactive in the first 24 h after PDT and this effect was maintained until 72 h. Meanwhile, PDT exerted a lower effect on MTS reduction at 72 h than at 24 h post-PDT (*p* < 0.001) at the fluences of 10 J/cm^2^ and 15 J/cm^2^, indicating that the PDT effect was attenuated over time. 

**Figure 5:**
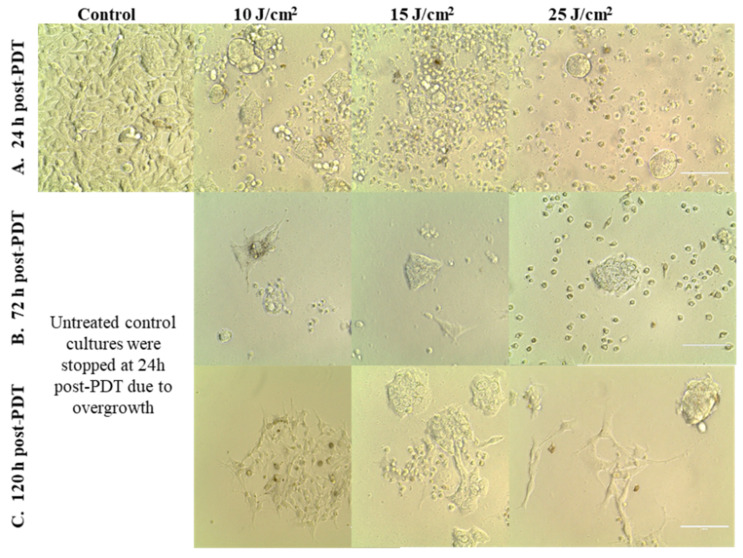
Images of HT29 tumor cells at various time points after PDT performed with various light fluences (10–25 J/cm^2^), which were delivered with the fluence rate of 50 mW/cm^2^. Cells were visualized by bright-field microscopy at 24 h (**A**), 72 h (**B**) and 120 h (**C**) post-PDT (100 µm scale bar).

This observation was sustained by microscopic investigations that showed the presence of adhered cells at 24 h and 72 h post-PDT, most probably viable cells, even in the case of the stronger PDT regimen of 25 J/cm^2^ (Figure 5A,B). While the number of adhered cells decreased over time, a few cells that appeared to be less affected by PDT continued to slowly proliferate and formed cell islets at 120 h post-PDT (Figure 5C). 

**Figure 6:**
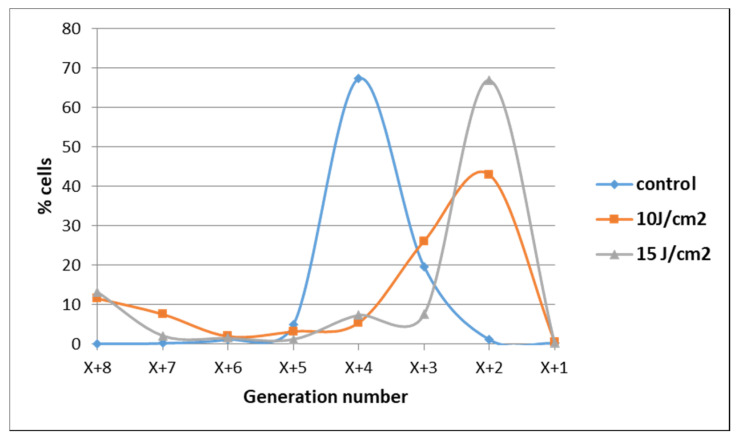
Demonstrative data on the proliferation of HT29 tumor cells subjected to PDT (fluences: 10 J/cm^2^ and 15 J/cm^2^; fluence rate: 50 mW/cm^2^) vs. non-treated control cells at 72 h post-PDT. Results are presented as a percentage of cells in various consecutive daughter generations in comparison with the parent population distanced by X previous generations. Data were obtained by flow cytometry with CFDA-SE and were processed using the ModFit LT software.

The observation that some tumor cells might have escaped from the deleterious action of PDT was also evidenced when cell proliferation was assessed at 72 h post-PDT by flow cytometry with CFDA-SE. Most of the cells exposed to 10 J/cm^2^ or 15 J/cm^2^ PDT were found at 72 h in lower-order daughter generations as compared to control cells, indicating that their overall proliferation was slower (Figure 6). Concurrently, a low cell percentage was found in higher-order daughter generations at 72 h post-PDT, suggesting that they were not harmed by PDT and actively proliferated at higher rates than control cells. These few cells seem to have gained a proliferation advantage.

#### 2.1.2. PDT-Induced Alteration of Membrane Integrity

**Figure 7:**
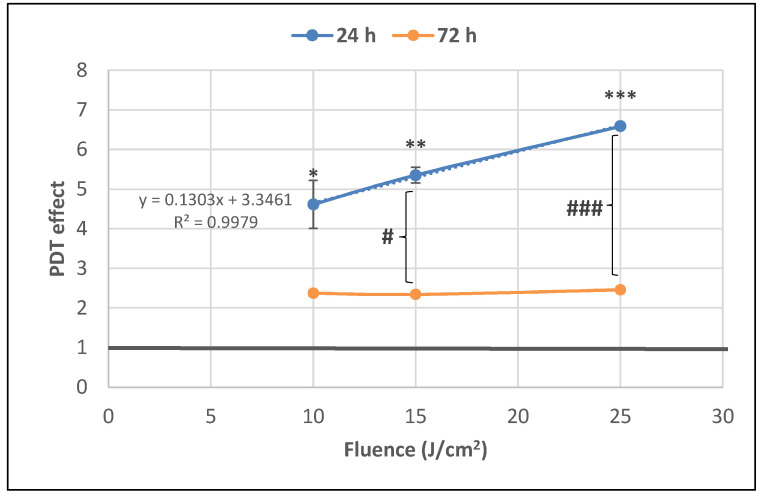
The dependence of LDH release by PDT-treated HT29 tumor cells on the fluence of the activating light delivered at a fluence rate of 50 mW/cm^2^. Analysis was performed at 24 h and 72 h post-PDT. Results are presented as PDT effect (mean ± SEM) in triplicate samples. PDT effect was calculated as OD in samples subjected to PDT divided by the mean value of OD in control samples. Comparison between samples was performed using Student’s *t*-test considering unequal variances: comparison between PDT regimens at a specific time-point: * *p* < 0.05, ** *p* < 0.01, *** *p* < 0.001; comparison between samples at 24 h and 72 h: # *p* < 0.05, ### *p* < 0.001.

The significant decrease of the number of metabolically active tumor cells registered at 24 h post-PDT (Figure 4) was associated with a fluence-dependent linear increase of LDH release, indicating alteration of the plasma membrane (Figure 7). The PDT-induced increase of LDH release was negatively correlated with the decrease of MTS reduction (Pearson r = 0.986, *p* = 0.106), following a linear regression equation (y = −0.09X + 0.64). Results indicated that, at least partly, the decrease of metabolically active tumor cells in PDT-treated samples was due to membrane alterations generally occurring in necrotic and necroptototic cells [9,10], which allow significant LDH release. 

The PDT effect on LDH release was lower at 72 h compared to 24 h for all the investigated light fluences and became fluence-independent in time (Figure 7). Results suggest that PDT induced persistent plasma membrane alterations in the time frame of 24–72 h post-PDT, especially in the first 24 h. 

**Figure 8:**
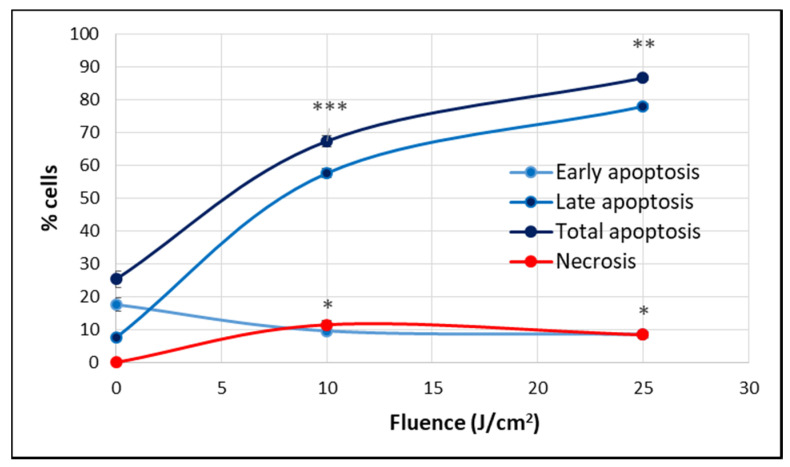
The percentage of apoptotic and necrotic HT29 cells in samples exposed to PDT (10 J/cm^2^ and 25 J/cm^2^, 50 mW/cm^2^) and in non-treated controls. Apoptosis and necrosis were assessed by flow cytometry using FITC-annexin V and propidium iodide at 24 h post-PDT. Results are presented as percentage (mean ± SEM) of cells in triplicate samples, regarding early, late and total apoptosis, as well as necrosis. Comparison between treated and non-treated samples was performed using Student’s *t*-test considering unequal variances. * *p* < 0.05, ** *p* < 0.01, *** *p* < 0.001.

At 24 h post-PDT, when the percentage of metabolically active cells decreased drastically below 50% in a fluence-dependent manner (Figure 4), many cells were found detached and rounded up, as seen in Figure 5A. For clarifying cell death, apoptosis and necrosis were investigated by flow cytometry with annexin V-propidium iodide. An important increase of the percentage of apoptotic cells, more specifically late apoptotic cells, accompanied by a smaller increase of necrotic cells was registered in PDT-treated vs. untreated cultures at 24 h post-PDT (Figure 8). 

**Figure 9:**
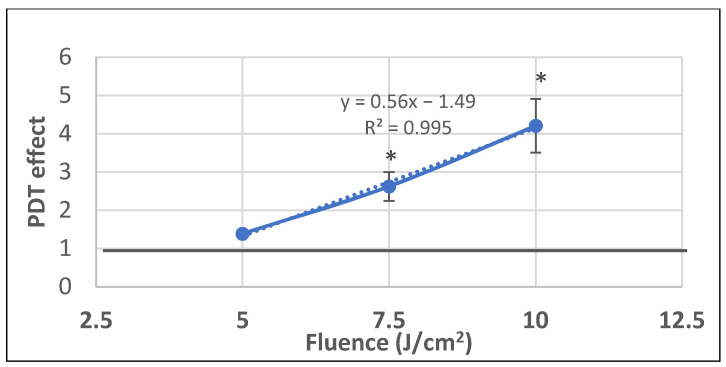
The dependence of LDH release by HT29 tumor cells exposed to milder PDT conditions on the fluence of the activating light (5–7.5–10 J/cm^2^) that was delivered at a lower fluence rate of 10 mW/cm^2^. Results are presented as PDT effect (mean ± SEM) in triplicate samples. PDT effect was calculated as OD in samples subjected to PDT divided by the mean OD value in controls. Comparison between samples was performed using Student’s *t*-test considering unequal variances: * *p* < 0.05.

We further investigated whether P2.2-PDT is also efficient in milder PDT conditions with lower light fluences (5–10 J/cm^2^) delivered at a lower fluence rate of 10 mW/cm^2^. A linear fluence-dependent increase of PDT effect on LDH release was registered at 24 h post-PDT, with significant differences between untreated controls and samples exposed to the higher investigated light fluences of 7.5 J/cm^2^ and 10 J/cm^2^ (Figure 9). Results emphasized once again that important damages at the level of plasma membrane, resulting in LDH release, were induced by PDT within 24 h post-treatment. Moreover, it appears that LDH release is not influenced by the fluence rate, as demonstrated by similar PDT effects on LDH release at 10 mW/cm^2^ and 50 mW/cm^2^ for a light fluence of 10 J/cm^2^ (PDT effect at 10 mW/cm^2^ was 4.2 ± 0.7 and at 50 mW/cm^2^ it was 4.6 ± 0.6). 

Altogether, results showed that, at least from the point of view of LDH release, which provides information on plasma membrane integrity, P2.2-PDT was efficacious on tumor HT29 cells in the light fluence domain of 7.5–25 J/cm^2^, with the highest damaging effects registered at 25 J/cm^2^. 

### 2.2. PDT-Induced Gene Expression Changes 

We have shown above that in approximately 24 h, more than 50% of HT29 tumor cells were affected by P2.2-PDT, despite the fact that an acute oxidative burst is known to be generated instantaneously in the course of PDT [11]. Possibly, tumor cells develop rescue mechanisms that delay cell death and may even protect some cells against the deleterious action of PDT (Figure 5C). 

Therefore, we investigated by qRT-PCR the expression profile of 84 genes (Table 1) that are critically involved in cellular responses to stressors such as oxidative stress, hypoxia, osmotic stress, genotoxic stress, unfolded protein response and inflammation, as well as in cell death by apoptosis, necrosis and autophagy. Briefly, cells exposed in vitro to PDT (10 J/cm^2^, 50 mW/cm^2^) were harvested 24 h post-PDT. The gene expression pattern was evaluated separately in adhered and detached cells as potentially living and apoptotic/necrotic cells, respectively. 

According to the data presented in Table 2, 43 genes were found differentially expressed in P2.2-treated cells compared to controls (1.5 < FC < −1.5, *p* < 0.05). Among these genes, 38 were upregulated and 5 were downregulated either in adhered or detached cells. Selected genes were classified according to their known association with various types of stress (oxidative stress, hypoxia, genotoxic and proteotoxic stress, along with inflammation), some of them being part of several signaling networks. 

#### 2.2.1. Oxidative Stress

**Figure 10:**
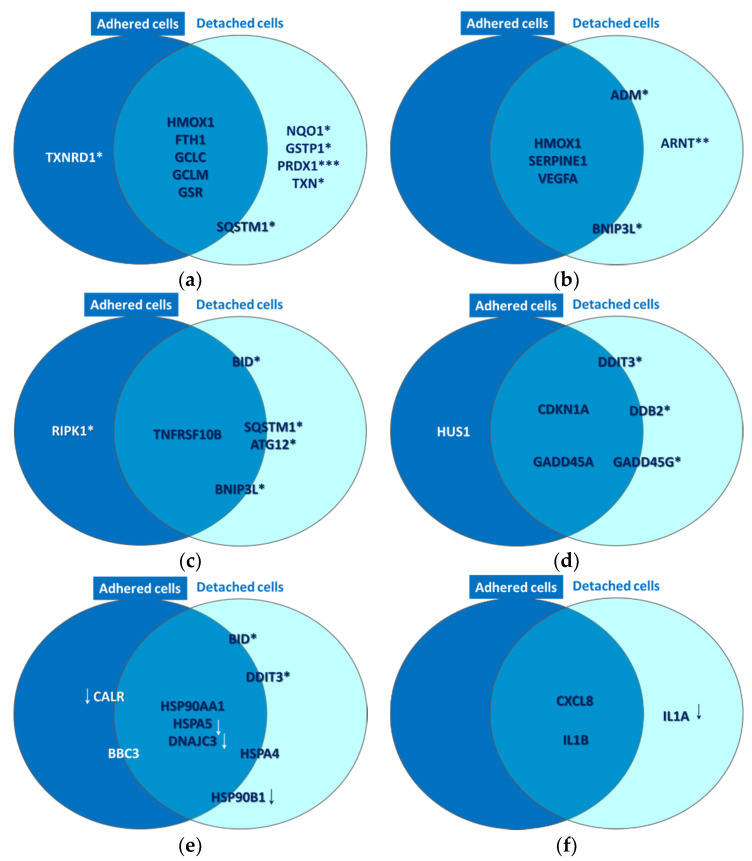
Common and distinctive gene expression patterns in adhered and attached HT29 tumor cells that were exposed to P2.2-PDT (10 J/cm^2^, 50 mW/cm^2^) at 24 h post-PDT. Some selected genes with modified expression in PDT-treated cells as compared to untreated cells (1.5 < FR < −1.5, *p* < 0.05) are represented. These genes are involved in (**a**) oxidative stress, (**b**) Hypoxia signaling, (**c**) cell death, (**d**) DNA damage, (**e**) unfolded protein response and (**f**) inflammation. All the reported genes were upregulated, excepting those marked with downwards arrows. Genes placed at the borders of the two circles are expressed both in adhered and detached cells, but have higher expression in one of the two cell types. This comparison between gene expression levels in adhered and detached cells was performed with a paired samples *t*-test (* *p* < 0.05, ** *p* < 0.01, *** *p* < 0.001).

A strong molecular fingerprint of oxidative stress was evidenced in PDT-treated samples by the upregulation of 11 redox genes involved in antioxidant defense (Table 2). The activation of antioxidant mechanisms demonstrated on the one hand that cells were indeed subjected to an oxidative challenge during PDT, as expected considering the PDT-generated burst of singlet oxygen [3,12]. On the other hand, gene expression data pointed out the upregulation of several protective redox genes that aim to counteract or delay cell death induced by the oxidative challenge generated by PDT. The identified antioxidant genes are involved in iron metabolism (*HMOX1*, *FTH1*) [13], in glutathione (*GCLC*, *GCLM*, *GSR*, *GSTP1*) [14] or thioredoxin (*TXN*, *TXNRD1*) [15] metabolism, as well as in other cytoprotective pathways (*PRDX1* [16], *NQO1* [17,18] and *SQSTM1* [19,20]). Some of these upregulated genes were common to adhered and detached cells, while other genes were preferentially upregulated either in detached or adhered cells (Figure 10a). Thus, *PRDX1* (*p* < 0.001), *NQO1* (*p* < 0.05), *GSTP1* (*p* < 0.05) and *TXN* (*p* < 0.05) were distinctively expressed in detached cells, while *SQSTM1* (*p* < 0.05) had a higher transcript level in detached cells as compared to adhered cells. Alongside this, *TXNRD1* (*p* < 0.05) was exclusively overexpressed in adhered cells. Altogether, results indicated that a robust antioxidant response is generated by PDT, which triggers antioxidant defense mechanisms that appear to be insufficient to counteract the deleterious action of PDT, resulting in the significant decrease of viable tumor cells (Figure 4, Figure 7 and Figure 8). Surprisingly, the protective antioxidant response was stronger in detached cells than in adhered cells (Table 2). This observation might be explained by a higher photosensitizer load in some cells (Figure 3), that possibly led to a stronger singlet oxygen burst triggered by PDT and, consequently, to robust activation of antioxidant mechanisms. 

#### 2.2.2. Hypoxia Signaling

Gene expression data indicated that P2.2-PDT induced the activation of hypoxia signaling, as evidenced by the upregulation of six pathway-relevant genes (Table 2), namely *SERPINE1* [21], *ADM* [22], *ARNT* [23], *VEGFA* [24] and *BNIP3L* [25]. Additionally, *HMOX1* [26], which is at the crossroad of oxidative stress, hypoxia and inflammation [27], was also found highly overexpressed. As shown in Figure 10b, *HMOX1*, *SERPINE1* and *VEGFA* were upregulated both in adhered and detached cells, *ARNT* was upregulated only in detached cells (*p* < 0.01), while the transcript levels of *BNIP3L* and *ADM* were increased in detached vs. adhered cells (*p* < 0.05). 

#### 2.2.3. Cell Death

As previously explained, P2.2-PDT was shown to induce significant apoptotic and necrotic cell death within 24 h post-treatment (Figure 8). We also found that several cell death-related genes were upregulated in PDT-exposed cells (Table 2), complementing the functional and phenotypic data on cell death following PDT (Figure 7 and Figure 8). The identified genes are involved in apoptosis (*TNFRSF10B* [28], *BID* [29], *BNIP3L* [25] and *BBC3* [30]), necrosis (*RIPK1* [31]) and autophagy (*ATG12* [32], *SQSTM1* [33]). As shown in Figure 10c, *TNFRSF10B* was found overexpressed both in adhered and detached cells. Meanwhile, *BID*, *ATG12*, *SQSTM1* and *BNIP3L* were preferentially expressed in detached cells (*p* < 0.05) and *RIPK1* (*p* < 0.05) in adhered cells, albeit not exclusively. This gene expression pattern at 24 h post-PDT suggests that distinctive death mechanisms might be activated in detached and adhered cells. Of note, death genes were shown to be overexpressed in adhered cells (presumably living cells), indicating that they were in fact committed to death that may occur sometimes after the first 24 h post-PDT.

In the context of cell death, we identified several genes that were found to be upregulated by P2.2-PDT, which are related to cellular responses to genotoxic or proteotoxic stress, as will be presented below. 

#### 2.2.4. DNA Damage

PDT-induced DNA damage was indirectly evidenced by the upregulation of eight genes involved in DNA damage repair mechanisms, such as cell cycle arrest (*CDKN1A* [34], *GADD45A*/*G* [35], *CHEK2* [36] and *HUS1* [37], or other DNA damage responses (*DDIT3* [38], *DDB2* [39] and *XPC* [40] (Table 2). As shown in Figure 10d, the *CDKN1A* and *GADD45A* genes involved in cell cycle arrest were found overexpressed in both adhered and detached cells. This finding suggests that the observed decrease of the number of metabolically active cells in PDT-treated samples (Figure 4) might be due not only to cell death (Figure 7 and Figure 8, Table 2), but also to a proliferation inhibition (Figure 6). *DDIT3*, *GADD45G* and *DDB2* had higher transcript levels in detached vs. adhered cells (*p* < 0.05), probably due to increased DNA damage in detached cells, while *HUS1* was moderately overexpressed only in adhered cells (FC = 1.62, *p* < 0.05). 

#### 2.2.5. Unfolded Protein Response

P2.2-PDT was shown to induce proteotoxic stress, probably due to the PDT-inflicted oxidative damage of proteins. Proof for ER stress and the consequent UPR was provided by significant expression changes of nine pathway-specific genes, out of which five genes were upregulated and four genes were downregulated (Table 2). Thus, *DDIT3*, which is induced by ER stress [41], along with *BBC3* and *BID*, which link the ER stress response to the mitochondrial apoptosis pathway [42], were concurrently found overexpressed. Some other genes encoding protective ER chaperones (*HSP90AA1* and *HSPA4*) were also found upregulated. They may target misfolded proteins for degradation and even shut down UPR when the stress subsides [43]. Meanwhile, some other chaperone-encoding genes, such as *HSP90AA1* [44], *HSPA5* [45] and *HSP90B1* [46], were found to be downregulated in PDT-treated cells, along with *CALR* [47] and *DNAJC3* [48]. Accordingly, important mechanisms in UPR might be suppressed in cells exposed to PDT, potentially leading in time to cell death. As shown in Figure 10e, while *HSP90AA1* was markedly overexpressed both in adhered and detached cells, *HSPA5* and *DNAJC3* were found concurrently downregulated in both types of cells. Meanwhile, the genes connecting ER stress and apoptosis (*DDIT3* and *BID*) were preferentially overexpressed in detached cells (*p* < 0.05), which also exhibited downregulation of the protective *HSP90B1* gene (Figure 10e). Surprisingly, *CALR* was downregulated by PDT mostly in adherent cells, thus making them more susceptible to ER stress and to its deleterious consequences. In turn, *CALR* downregulation may have beneficial effects in tumors, considering that this can induce inhibition of cell growth, invasion and cell cycle progression [49]. 

#### 2.2.6. Inflammation

A pro-inflammatory cytokine response was shown to be elicited by PDT in HT29 tumor cells, as seen from the significant overexpression of the pro-inflammatory *CXCL8* and *IL1B* genes in both adhered and detached cells (Table 2, Figure 10f). Surprisingly, *IL1A*, encoding for a danger signal that senses genotoxic stress and is released by cells with damaged plasma membrane [50], was found distinctively downregulated in detached cells (*p* < 0.05) as shown in Table 2 and Figure 10f. Possibly, *IL1A* transcription was inhibited after the activation of the apoptotic machinery, cells having already sensed the death-inducing signals.

## 3. Discussion

The study brought first experimental in vitro proof on the photosensitizing ability of the new asymmetric porphyrinic compound P2.2 [8] for efficient PDT in the human colon carcinoma HT29 cell line. Following P2.2-PDT, we showed that tumor cells massively died by necrosis and apoptosis within 24 h, especially at high activating light fluences of 25 J/cm^2^, as demonstrated using functional and phenotypic tests. 

Gene expression data highlighted the molecular mechanisms underlying cell death and survival following a milder P2.2-PDT regimen (10 J/cm^2^). Thus, the observed increased apoptosis of PDT treated-cells might be partly mediated by the Death receptor 5 (DR5), also known as TRAIL receptor 2 (TRAILR2) or tumor necrosis factor receptor superfamily member 10B (TNFRSF10B), which triggers the extrinsic apoptotic cascade [51]. DR5 has been found upregulated in various types of tumors, including colorectal carcinomas, and can be exploited for selective apoptotic killing of cancer cells through caspase 8 [52]. Recent studies have shown that PDT extensively sensitizes refractory colon tumors to death signals delivered by long-acting TRAIL [53], possibly by increasing the levels of TRAIL receptors on tumor cells. Furthermore, the increased levels of the *BID* transcripts evidenced by us may sustain the connection of death receptor signaling to the mitochondrial apoptotic machinery [54]. The activation of the mitochondrial apoptotic pathway in PDT-treated cells is also demonstrated by the increased transcript levels of the *BBC3* gene which is under TP53 transcriptional control but can be independently upregulated also by ER stress [55]. A complementary death mechanism, which is highly relevant for tumors and for PDT [56], was evidenced by the overexpression of the *BNIP3L* gene, which is associated with TP53-dependent apoptosis under hypoxia [25]. 

A particular death pathway bridging apoptosis and necrosis (necroptosis or programmed necrosis) might occur in PDT, as evidenced by *RIPK1* overexpression [57,58]. It is known that necroptosis and necrosis can initiate inflammatory reactions, albeit through distinctive mechanisms, and this may defend or sustain tumor progression, depending on the context [57]. Finally, we have found that autophagy might also be activated by P2.2-PDT, as shown by *ATG12* and *SQSTM1* upregulation, indicating that damaged cells attempt to tolerate the photo-damage or are partly driven to autophagic cell death [59].

We have evidenced that PDT-triggered genotoxic stress may drive tumor cells towards death. In turn, PDT-treated cells responded by upregulating genes involved in cell cycle arrest and in other DNA damage responses (DDR) as defense mechanisms against DNA injury [60]. PDT-induced DNA damage, accompanied by the failure of DNA repair mechanisms, can result in the death of a high percentage of the treated cells [61], as also shown by our cell viability data. Most interesting, among the overexpressed genes involved in DDR, we identified genes with dual roles, which can facilitate DNA repair or induce apoptosis, depending on the context. For instance, the multifunctional DDB2 protein (damaged DNA binding protein 2) is known to contribute to DNA repair through the induction of nucleotide excision repair [62], but can also drive damaged cells towards apoptosis [63,64], for instance by downregulating *CDKN1A* expression [65]. Moreover, it has been shown that DDB2 can regulate the transcription of the antioxidant *SOD2* gene, hence regulating superoxide levels and consequently the redox balance [66]. 

Molecular analysis of cell death revealed that PDT triggered a complex web of stressors that may lead either to cell death or to resistance of treated tumor cells, as detailed below. We have emphasized that, in time, the few cells surviving PDT continued to grow slowly, probably accounting for disease relapse sometimes after PDT. Therefore, unraveling the rescue mechanisms triggered by PDT may be of highest importance for designing new adjuvant therapeutic strategies aimed at sustaining PDT.

P2.2-PDT induced a strong oxidative burst that was indirectly evidenced in this study through the upregulation of several potent antioxidant genes. Surprisingly, a robust antioxidant response was generated by PDT not only in detached cells (apoptotic cells) but also in adhered cells (presumably living cells), and this protective response was even stronger in detached cells. This observation might be explained by a potentially higher photosensitizer load in some cells, probably resulting in stronger PDT-induced singlet oxygen burst and extensive activation of antioxidant mechanisms. Nonetheless, the elicited antioxidant response was not sufficiently protective to completely counteract the observed PDT-driven decrease of viable cells number. In turn, the remaining viable cells may get shielded against oxidative damage and become resistant to future PDT sessions as well as to other anticancer therapies that rely on oxidative stress for cytotoxicity [3]. 

The hypoxia signature detected in this study in PDT-treated cells sustains the assumption that the acute oxidative burst triggered by PDT can transiently reduce local oxygen availability due to its utilization for singlet oxygen generation [3]. PDT-induced hypoxia adds to the intrinsic hypoxia of large tumors [67]. Through a feedforward loop, acute hypoxia can induce within minutes a superoxide burst that intensifies the oxidative stress triggered by PDT through the generated singlet oxygen [68]. It has been also shown that reactive oxygen species (ROS) can directly stabilize and activate the transcriptional activity of the hypoxia-inducible factor-1 alpha (HIF1α), which regulates the expression of many hypoxia-responsive genes [69]. Unfortunately, hypoxia signaling is generally associated with increased angiogenesis and epidermal to mesenchymal transition (EMT), both processes favoring tumor progression [70]. Therefore, besides being cytotoxic against most of the treated tumor cells, the PDT regimen investigated by us appears also to support the survival of residual cancer cells that escaped from the therapeutic hit through hypoxia signaling.

The accumulation of oxidatively damaged proteins resulting from the severe oxidative burst triggered by PDT may account for the ER stress signature and the consequent UPR detected by us at transcriptional level in PDT-treated cells [71]. Alternatively, we cannot rule out that ER stress might be induced directly by PDT if the photosensitizer localizes, at least partly, in ER, and a massive amount of ROS is produced locally [72]. Considering the high reactivity of photo-generated ROS, autophagy was shown by us to be initiated to remove damaged organelles [73], if cells were not driven to apoptosis by DDIT3 in conjunction with BID and BBC3 [59,74,75]. Additionally, we evidenced the upregulation of the heat shock protein-encoding genes *HSP90AA1* and *HSPA4*, which may limit the PDT-induced proteotoxic stress. In turn, other protective genes were found downregulated (*HSPA5*, *DNAJC3*, *HSP90B1* and *CALR*), indicating that important cytoprotective mechanisms might have been suppressed by the investigated PDT regimen. 

As will be detailed below, several transcription factors regulate the expression of the genes found significantly modified in the present study. 

All the investigated redox genes are known targets of the transcription factor NRF2 which controls at the transcriptional level more than 250 cytoprotective genes, most of them having an antioxidant role [76]. It has been shown that singlet oxygen, the peculiar form of ROS generated during PDT, can activate the NRF2 system directly or via derived oxidized products, therefore inducing antioxidant shielding against the PDT-inflicted injuries. Moreover, NRF2 links oxidative conditions with DDR through the p21 protein which is encoded by *CDKN1A* and is mainly involved in cell cycle arrest [34]. NRF2 can induce the transcription of the *CDKN1A* gene which is also under the transcriptional control of TP53 [77]. Through a feed forward loop, enhanced levels of p21 compete with NRF2 for binding to its KEAP1 repressor, hence inducing the activation of the NRF2 pathway [78]. Altogether, NRF2 silencing would be a promising co-therapy for increasing PDT efficacy [79] but, for the moment, there are no NRF2 inhibitors approved for therapeutic use, due to their numerous off-target effects [80]. 

**Figure 11:**
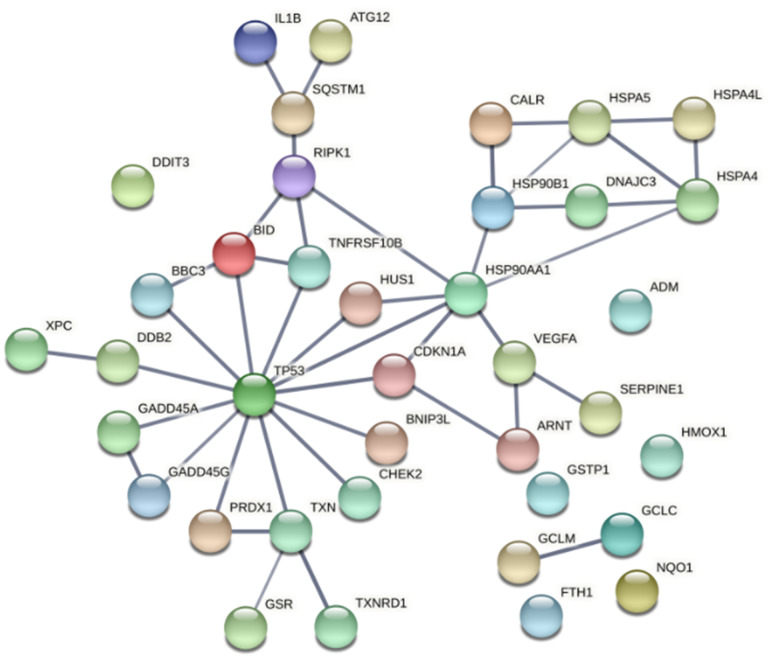
Functional associations between the tumor suppressor TP53 and the proteins encoded by genes with modified expression in PDT-treated HT29 tumor cells, as compared to non-treated cells (Table 2). The network was built using the online tool “STRING: functional protein association network”. Only connections deriving from experiments and databases, with a high confidence of 0.7, are represented.

Most of the proteins encoded by the genes identified in this study are directly or indirectly connected to the redox-sensitive tumor suppressor TP53 (Figure 11). TP53 is one of the most important genome guardians that protects cells against various insults, including oxidative stress, by driving damaged cells towards death or by activating rescue mechanisms [81,82,83]. Interestingly, it has been shown that TP53 might play a significant role in the death of cells subjected to porphyrin-PDT, through direct interaction with the drug itself, leading to induction of TP53-dependent cell death both in the dark and upon PDT [81]. Nevertheless, our data also point towards a TP53-mediated activation of cytoprotective mechanisms that might render treated cells resistant to future PDT sessions. It is worth mentioning that many genes that are known to be under TP53 control can be also upregulated by other transcription factors, depending on the stressors to which cells were exposed. Therefore, the involvement of TP53 has to be carefully analyzed in PDT for finding the proper therapy regimen that drives tumor cells towards death.

The PDT-mediated upregulation of some gene targets of the redox- and hypoxia-sensitive transcription factor HIF1α, such as *VEGFA*, give evidence that a hypoxia response is indeed triggered by PDT. HIF1α may elicit protective mechanisms that not only defend tumor cells against PDT, but may further drive tumor progression by sustaining angiogenesis and EMT [84,85]. 

A transcription factor revealed by our results to be transcriptionally upregulated by P2.2-PDT is DDIT3 (CHOP). This stress-induced transcription factor has been shown to integrate the signals from multiple stressors such as DNA damage and ER stress. It mediates a vast array of cellular responses, from UPR to stress-induced cell death, either per se or through interaction with other transcription factors [38]. As evidenced in this study, *DDIT3* overexpression was paralleled by the upregulation of the *TNFRS10B* gene that encodes the death receptor DR5. This might be a functional association between cell death and ER stress, considering that *TNFRS10B* transcription is under the control of DDIT3, in addition to TP53 and NFκB [86,87]. Moreover, it has been shown that DR5 along with other TRAIL receptors serve as stress-associated molecular patterns (SAMPs) to promote ER stress-induced inflammation [88]. Besides the involvement of DDIT3 in apoptotic and autophagic cell death [75], it has been demonstrated that this transcription factor can activate inflammatory responses that generally sustain tumor progression [89]. This can explain, at least partly, some of our results regarding the PDT-induced inflammatory response in HT29 colon carcinoma cells. For instance, we detected in PDT-treated cells a markedly enhanced transcription of the *CXCL8* gene encoding the IL-8 chemokine and this might be mediated by DDIT3 [90]. IL-8 triggers enhanced recruitment of neutrophils in the tumor niche [91], their cytotoxic activity increasing PDT efficacy against tumor cells [92], hence limiting disease progression [93,94]. In turn, increased levels of IL-8 were shown to boost colorectal liver metastasis, hence worsening disease outcome [95]. We do not rule out that enhanced *CXCL8* expression could derive also from NFκB-mediated gene transcription under the PDT pressure [96]. 

Altogether, molecular data highlighted that the investigated PDT regimen triggered increased transcription of critical genes that underlies the therapeutic effect, but some of the investigated genes may confer a survival advantage to tumor cells. The differential analysis of the gene expression pattern in adhered and detached cells could not provide definite evidence on the particular genes that determine the difference between tumor cells killed by PDT and the surviving ones at 24 h post-PDT. Most of the selected genes were common to adhered and detached cells, indicating that adhered cells might be in fact committed to delayed death. Only *TXNRD1* and *RIPK1* were upregulated specifically in adhered cells, being involved in antioxidant protection and cell death, respectively. The antioxidant genes *NQO1*, *GSTP1*, *PRDX1* and *NQO1* were upregulated specifically in detached cells, indicating that antioxidant mechanisms were not fully capable of protecting tumor cells against the PDT-inflicted oxidative injuries. 

## 4. Materials and Methods

### 4.1. Photosensitizer 

The unsymmetrical porphyrin 5-(4-hydroxy-3-methoxyphenyl)-10,15,20-tris-(4-acetoxy-3-methoxyphenyl)porphyrin (Figure 1), abbreviated as P2.2, was obtained according to the method previously described by us [8]. The method is based on the interaction between 4-hydroxy-3-methoxybenzaldehyde, pyrrol and 4-acetoxy-3-methoxybenzaldehyde (3:1 ratio) for approximately 3 h in an acid environment, under strict temperature control (t = 125 °C). P2.2 purification was obtained by column chromatography using Al_2_O_3_ 90 (Merck, 63–200 μm and 70–230 μm mesh) as stationary phase and dichloromethane/diethyl ether (30:1 *v*/*v*) as eluent. The synthesis yield was 7% and the purity was 100%, as previously shown by NMR analysis. The fluorescence emission and singlet oxygen formation quantum yields, lifetimes and the methods used for quantification are described in [8] and [97]. P2.2 has a Q band with a peak at 628 nm and was therefore activated for PDT using a 635 nm laser in the Modulight ML6600 equipment (Modullight, Tampere, Finland). The obtained P2.2 was dissolved at 10 mM concentration in PEG 200, a biocompatible solvent, and was stored at room temperature in the dark until use.

### 4.2. Cells

The human colon adenocarcinoma cell line HT29 purchased from the American Tissue and Cell Collection (ATCC^®^ HTB-38™, Manassas, VA, USA) was used. Cells were grown in DMEM-F12 culture medium with GlutaMAX (Gibco), supplemented with 10% fetal bovine serum (FBS, Sigma, Saint Louis, MO, USA), that will be hereinafter referred to as complete culture medium. Twice per week cells were detached with 0.05%/0.02% (*w*/*v*) Trypsin-EDTA (Biochrom). After trypsin inactivation with two volumes of complete culture medium, cells were washed by centrifugation (1200 rpm, 5 min, 4 °C), were suspended in complete culture medium and were counted by optical microscopy in a Burker–Turk counting chamber, using Trypan blue as dead cells stain. Only cell cultures with a viability >95% were used for experiments. For multiplication, cells were seeded in 25 cm^2^ cell culture flasks (40,000 live cells/cm^2^) and were cultivated at 37 °C in 5% CO_2_ atmosphere.

### 4.3. Loading of Cells with P2.2 

HT29 cells (0.5 × 10^6^ cells) were seeded in 35 mm Petri dishes in 2 mL complete culture medium, in triplicates for each experimental condition. Cells were cultivated for 24 h in 5% CO_2_ atmosphere at 37 °C for allowing their adherence. Thereafter, the complete culture medium was discarded and was replaced with 2 mL DMEM-F12 culture medium with GlutaMAX, supplemented with 2% FBS and 10 µM P2.2 (referred as P2.2-loading culture medium). Cells were cultivated for another 24 h to allow P2.2 loading. All the procedures with P2.2 and P2.2-loaded cells were performed in “dark” conditions (no direct light falling on the samples) for avoiding uncontrolled activation of the photosensitizer. 

### 4.4. P2.2 Uptake in HT29 Cells 

For measuring P2.2 uptake into HT29 cells, 0.1 × 10^6^ cells were seeded in triplicates in 24 well plates and were cultivated for 24 h in 0.5 mL complete culture medium for allowing their adherence. The culture medium was then discarded and was replaced with P2.2-loading culture medium (see Section 4.3). At 24 h after P2.2 addition to cell cultures, cells were detached with Trypsin-EDTA (see Section 4.2), were washed by centrifugation and were finally suspended in Live Cell Imaging Solution (Thermo Fisher Scientific, Waltham, MA, USA). The uptake control samples were not incubated with P2.2 and were processed exactly as P2.2-treated cells. All the procedures with P2.2-loaded cells were performed in “dark” conditions. P2.2 incorporation into HT29 cells was measured based on P2.2 red fluorescence by flow cytometry on a BD FACSCanto II cytometer with BD FACSDiva 6.1 software (Becton Dickinson, Franklin Lakes, NJ, USA). Data from a minimum of 10,000 events were acquired. Fluorescence was expressed in arbitrary units. Fluorescence data were processed in each sample as median fluorescence value or fluorescence distribution, using the above-mentioned software. 

### 4.5. In Vitro PDT 

After loading of cells with P2.2 in 35 mm Petri dishes (see Section 4.3), the P2.2-loading culture medium was discarded and cells were gently washed with complete culture medium at room temperature. Two mL Hank’s balanced salt solution supplemented with 2% FBS (PDT culture medium) was added and cells were subjected to in vitro PDT. PDT was performed in test samples at room temperature, using a Modulight ML6600 instrument (Modulight, Tampere, Finland) equipped with a 635 nm laser, illumination chamber for 35 mm Petri dishes and software control of temperature and PDT parameters (light fluence, fluence rate, power and time). The PDT parameters applied in various experimental settings were: 5, 7.5, 10, 15 and 25 J/cm^2^. Light was delivered at a fluence rate of 50 mW/cm^2^. A comparison between 10 and 50 mW/cm^2^ fluence rate was performed for a fluence of 10 J/cm^2^. Control samples loaded with P2.2 were prepared by applying the same procedure as described above, were not subjected to PDT and were kept at room temperature during the time when test samples were exposed to PDT. Immediately after PDT, the PDT culture medium was removed and was replaced with 2 mL complete culture medium. Both test samples and controls were further cultivated in 5% CO_2_ atmosphere at 37 °C for performing various post-PDT investigations.

### 4.6. Post-PDT Investigations

#### 4.6.1. Preparation of Samples for Post-PDT Investigations

- Cell culture supernatants were harvested from samples cultivated for 24 h post-PDT and were centrifuged for eliminating detached cells. These cell-free culture supernatants were used for the LDH release assay at 24 h post-PDT. Cellular sediments resulting following centrifugation were suspended in a small volume of complete culture medium. Parts of these cells were used for post-PDT investigations at 24 h and parts for cell cultures were analyzed at 72 h post-PDT, as will be described below.
- Adhered cells were detached at 24 h post-PDT with Trypsin-EDTA (see Section 4.2). The resulting cell suspension was centrifuged and the sediment was suspended in complete culture medium. Parts of these cells were used for investigations at 24 h post-PDT and parts were plated for cell cultures to be analyzed at 72 h post-PDT, as will be described below.
- Detached and adhered cells harvested at 24 h post-PDT were mixed. Cells in non-treated samples were counted and the volume containing 10,000 control cells was calculated. For MTS reduction and LDH release (see below Section 4.6.2 and Section 4.6.3, respectively), the previously calculated cell suspension volume was collected from all samples, both PDT-treated and non-treated, was placed in 96 well plates and the culture volume was adjusted to 100 µL complete culture medium in each well. Triplicate samples containing only culture medium constituted the background control for these colorimetric tests. Cell cultures were incubated for another 48 h at 37 °C in 5% CO_2_ atmosphere and were analyzed at 72 h post-PDT.

#### 4.6.2. MTS Reduction

MTS reduction was used for evaluating the relative number of metabolically active cells in PDT-treated and non-treated samples. The method therefore provides information on cell viability and proliferation [8]. 

Detached and adhered cells harvested at 24 h post-PDT (see Section 4.6.1) were mixed for each sample. Cells in non-treated samples were counted and the volume containing 30,000 cells was calculated. This volume of cell suspension was harvested from all samples, both PDT-treated and non-treated, was adjusted to 100 µL and the resulting samples were analyzed for MTS reduction at 24 h post-PDT. MTS reduction was tested in the cell cultures prepared for 72 h investigations, as follows. A total of 50 µL of supernatant was collected from the cell cultures (see Section 4.6.1) following centrifugation of the 96 well plates at 150 g for 5 min and was used for assessing LDH release (see Section 4.6.3). Then, 50 µL of fresh complete culture medium was added to cell cultures for assessing MTS reduction.

MTS reduction was measured using the colorimetric CellTiter 96^®^ AQueous One Solution Cell Proliferation Assay (MTS) from Promega (Madison, WI, USA), according to the manufacturer’s procedure. Briefly, 20 µL of the kit’s reagent was added to each well and samples were cultivated 2 h at 37 °C for allowing MTS reduction by metabolically active cells. Finally, the optical density at 490 measured against a 620 nm wavelength reference was measured in each sample using a Sunrise Tecan microplate reader equiped with universal reader control and Magellan data analysis software (Tecan, Männedorf, Switzerland). Data were processed as corrected optical density (OD) obtained by subtrating the mean optical density of the background samples from the the optical density of test samples. Data were processed as PDT effect calculated by dividing the OD of each PDT-treated sample to the mean value of the OD of the corresponding control samples (not treated by PDT). 

#### 4.6.3. LDH Release

LDH release was used for evaluating membrane integrity of PDT-treated and non-treated cells [8]. The method provides information on cell death through necrosis/necroptosis [9]. 

Cell-free supernatants obtained immediately after PDT and at 24 h or 72 h post-PDT were tested for LDH release. 

LDH release was measured using the CytoTox 96^®^ Non-Radioactive Cytotoxicity Assay (Promega). The colorimetric test was performed according to the manufacturer’s procedure. Briefly, 50 µL of cell-free supernatant and 50 µL of LDH substrate were incubated in the dark at room temperature for 30 min. The reaction was interrupted by the addition of 50 µL stop solution. Finally, the optical density at 490 nm was measured in each sample using a Sunrise Tecan microplate reader equipped with universal reader control and Magellan data analysis software (Tecan). Data were processed as corrected optical density (OD) obtained by subtracting the mean optical density of background samples from the optical density of test samples. Data were processed as PDT effect calculated by dividing the OD of each PDT-treated sample to the mean OD value of control samples (not treated by PDT). 

#### 4.6.4. Apoptosis and Necrosis Evaluation by Flow Cytometry

The percentage of apoptotic and necrotic cells in P2.2-treated samples and in untreated controls was evaluated at 24 h post-PDT by flow cytometry using the Annexin A5 Apoptosis Detection Kit (BioLegend, San Diego, CA, USA). The test was performed according to the procedure indicated by the manufacturer. 

Briefly, detached and adhered cells harvested at 24 h post-PDT (see Section 4.6.1) were mixed for each samples. Cells were counted and approximately 300,000 cells from each sample were harvested. Cells were washed once with phosphate-buffered saline (PBS), were suspended in 100 µL annexin V binding buffer and were labelled with 5 µL FITC Annexin V and 10 µL of propidium iodide solution. Cell suspensions were incubated for 15 min at room temperature, in the dark. The reaction was stopped by the addition of 400 µL annexin V binding buffer. Samples were analyzed within 30 min by flow cytometry on a BD FACSCanto II cytometer with BD FACSDiva 6.1 software (Becton Dickinson). Data from a minimum of 5000 events were acquired. For PE-FITC the compensation was set at 31, while for FITC-PE it was set at 5. Flow cytometry data were presented as percentage of early apoptotic cells (annexin V^+^/PI^−^), late apoptotic cells (annexin V^+^/PI^+^) and necrotic cells (annexin V^−^/PI^+^). The total pecentage of apoptotic cells was calculated as sum of early and late apoptotic cells percentages. 

#### 4.6.5. Cell Proliferation Evaluation

Cell proliferation was assessed by flow cytometry with CFDA-SE (Vybrant^®^ CFDA SE Cell Tracer Kit, Invitrogen, Waltham, MA, USA). According to the information provided by the supplier, CFDA-SE passively diffuses into cells. It is colorless and nonfluorescent until its acetate groups are cleaved by intracellular esterases to yield highly fluorescent, amine-reactive carboxy-fluorescein succinimidyl ester. The succinimidyl ester group reacts with intracellular amines, forming fluorescent conjugates that are well-retained within cells. The dye–protein adducts that form in labeled cells are retained by the cells throughout development, meiosis and in vivo tracing. The label is inherited by daughter cells after cell division and is not transferred to adjacent cells in a population, therefore providing reliable information on cell proliferation. 

Adhered cells harvested at 24 h post-PDT (see Section 4.6.1) were counted. From each sample 300,000 cells were collected and were washed twice in cold PBS (1200 rpm, 5 min, 4 °C). Sedimented cells were incubated for 15 min with 5 µM CFDA-SE in 300 µL pre-warmed PBS (37 °C). Cells were washed once in PBS by centrifugation. Sedimented cells were suspended in 500 µL fresh complete culture medium and were incubated for another 30 min at 37 °C to ensure stable loading of cells. The suspension was washed by centrifugation and the cell pellet was suspended in 300 µL complete culture medium. Then, 100 µL of cell suspension from each sample was placed in 24 well plates and the culture volume was adjusted to 1 mL. Cell cultures were incubated for another 48 h at 37 °C in 5% CO_2_ atmosphere and were analyzed at 72 h post-PDT. Before investigation, cells were detached with Tripsin-EDTA solution (see Section 4.2) and were washed twice in ice-cold PBS. The intracellular fluorescence of CFDA-SE was measured by flow cytometry using a BD FACSCalibur flow cytometer and CellQuest Pro software (Becton Dickinson, Franklin Lakes, NJ, USA). CFDA-SE fluorescence was activated with a 482 nm laser, while emmission was registered in the FL-1 channel for fluorescein isothiocyanate (FITC). The data from a minimum of 50,000 events were acquired. Flow cytometry data were further processed using the ModFit LT software (Verity Sotware House, Topsham, ME, USA), which provides the distribution of proliferative cells in consequtive daughter generations.

#### 4.6.6. Microscopic Monitoring of Cell Cultures

Cell cultures were monitored in time, up to 120 h post-PDT, by optical microscopy using an EVOS XL Core Cell Imaging System equiped with image acquisition software (ThermoFisher Scientific). Of note is that separate cell cultures were prepared for imaging, equivalent to those described at 4.5, considering that exposure of P2.2-loaded cells to visible light might trigger an artificial activation of PDT and might compromise the results of the post-PDT tests. Due to the same reason, even the samples dedicated to imaging were only rarely subjected to microscopic evaluation (1 time/day, with only short exposure to light).

#### 4.6.7. Gene Expression

Cell cultures in 35 mm Petri dishes were prepared and processed as described in Section 4.3, Section 4.4 and Section 4.5. 

At 24 h post-PDT, cell supernatants containing detached cells were collected and centrifuged and cellular sediments were finally collected in 1 mL RiboZol reagent (VWR, Radnor, PA, USA). Attached cells were gently washed with warm PBS (37 °C) and 1 mL RiboZol reagent was then added for RNA extraction. Processed samples in RiboZol reagent were stored at −80 °C until use.

Total RNA isolation from cells preserved in RiboZol reagent (VWR Life science) was performed according to the manufacturer’s instructions. RNA concentration was quantified using the Nanodrop 2000 (Thermo Fisher Scientific, Waltham, MA, USA). Both the 260/280 nm and 260/230 nm ratios were above 1.8, indicating a high RNA quality. cDNA synthesis was performed using 600 ng of total RNA with the RT^2^ First Strand Kit (Qiagen, Hilden, Germany), according to the manufacturer’s instructions. The expression of 84 genes involved in stress and toxicity was assessed using the RT² Profiler™ PCR Array Human Stress and Toxicity PathwayFinder (PAHS-003Z) from Qiagen Table 1), using the SYBR Green chemistry on ABI7500 Fast PCR System (Thermo Fisher Scientific, Waltham, MA, USA). The expression level of each gene was normalized on the geometric mean values of two housekeeping genes (ACTB and HPRT1), which were selected according to RefFinder algorithm [98] after the analysis of five candidate reference genes (ACTB, B2M, GAPDH, HPRT1 and RPLP0) both in PDT-treated samples and non-treated controls. Gene expression data were analyzed with the RT^2^ Profiler PCR Array software package (Qiagen). Gene expression levels were calculated as 2^−ΔCT^ values. Fold change (FC) in gene expression was calculated as the 2^−ΔCT^ mean values in patients divided by 2^−ΔCT^ mean values in controls. Results are presented as fold regulation (FR) as follows: when the FC value was above 1, FR was equal to FC and results were reported as fold upregulation; when the FC value was less than 1, FR was expressed as the negative inverse of FC and results were reported as fold downregulation.

### 4.7. Data Processing

Whenever possible data were presented as mean value ± standard error of the mean (SEM) for triplicate samples. The IC50 value of P2.2 was computed using the online Quest Graph™ IC50 Calculator (https://www.aatbio.com/tools/ic50-calculator, accessed on 27 June 2021) and was expressed as mean ± standard deviation (SD). PDT effect was calculated as a parameter value in PDT-treated samples divided by the mean parameter value in non-treated controls. Supraunit values of PDT effect indicate activation, while subunit values indicate an inhibition. Comparison of the phenotypic and functional data from cells exposed to PDT and from non-treated controls was performed in Excel using the Student’s t-test (unequal variances or paired two samples for mean, depending on the case). Comparison of gene expression data between PDT-treated samples and controls was performed with the Student’s t-test (equal variances) using the RT^2^ Profiler PCR Array software package (Qiagen, Hilden, Germany). Comparison between adhered and attached cells regarding gene expression (FR values) was performed in Excel using the Student’s t-test (paired two samples for mean). Differences were considered significant for *p* values < 0.05. The Pearson test was used for evaluating correlations between parameters, considered as significant for r values > 0.6 and *p* < 0.05. 

## 5. Conclusions

Using an integrative systems biology approach on the expression of stress genes in HT29 human colon carcinoma cells subjected to porphyrin-PDT, the study highlighted the network of molecular mechanisms underlying therapy-induced cell death and other cellular responses to the complex web of stressors triggered concomitantly by P2.2-PDT, encompassing oxidative stress, hypoxia, DNA damage, ER stress and UPR, along with inflammation. The transcription factors potentially responsible for the observed gene expression profile (NRF2, HIF1α, p53, DDIT3 and, possibly, NFκB) were highlighted. Particular cytoprotective mechanisms and upregulated pathway-specific genes were found, which represent promising therapeutic targets for improving PDT efficacy. Nevertheless, this study does not provide information on the impact of the observed gene expression changes at protein and functional level, an aspect that should be addressed in future studies. The identified expression profile of stress genes triggered by the particular P2.2-PDT regimen used in the present study might be further extended to other porphyrinic photosensitizers and PDT regimens.

## Figures and Tables

**Table 1:** The list of analyzed genes, contained in the RT² Profiler™ PCR Array Human Stress and Toxicity PathwayFinder (Qiagen, PAHS-003Z).

**Oxidative Stress** FTH1, GCLC, GCLM, GSR, GSTP1, HMOX1, NQO1, PRDX1, SQSTM1, TXN, TXNRD1
**Hypoxia Signaling ** ADM, ARNT, BNIP3L, CA9, EPO, HMOX1, LDHA, MMP9, SERPINE1 (PAI-1), SLC2A1, VEGFA
**Osmotic Stress** AKR1B1, AQP1, AQP2, AQP4, CFTR, EDN1, HSPA4L (OSP94), NFAT5, SLC5A3
**Cell Death** *Apoptosis:* CASP1 (ICE), FAS, MCL1, TNFRSF10A (TRAIL-R), TNFRSF10B (DR5), TNFRSF1A (TNFR1). AutophagyATG12, ATG5, ATG7, BECN1, FAS, ULK1 *Necrosis:* FAS, GRB2, PARP1 (ADPRT1), PVR, RIPK1, TNFRSF10A (TRAIL-R), TNFRSF1A (TNFR1), TXNL4B
**DNA Damage and Repair** *Cell Cycle Arrest and Checkpoints:* CDKN1A (p21CIP1, WAF1), CHEK1, CHEK2 (RAD53), DDIT3 (GADD153, CHOP), HUS1, MRE11, NBN, RAD17, RAD9A *Other DNA Damage Responses:* ATM, ATR, DDB2, GADD45A, GADD45G, RAD51, TP53 (p53), XPC
**Unfolded Protein Response** ATF4, ATF6, ATF6B, BBC3 (PUMA), BID, CALR, DDIT3 (GADD153, CHOP), DNAJC3, HSP90AA1, HSP90B1, HSPA4 (HSP70), HSPA5 (GRP78)
**Inflammatory Response** CCL2 (MCP-1), CD40LG, CRP, CXCL8 (IL8), IFNG, IL1A, IL1B, IL6, TLR4, TNF

**Table 2:** Genes with statistically significant expression changes (1.5 < FR < −1.5, *p* < 0.05) in PDT-treated cells (10 J/cm^2^, 50 mW/cm^2^), either adhered or detached, as compared to untreated controls. Gene expression levels were assessed at 24 h post-PDT. Genes were classified according to the type of stress in which they are involved. Some genes are common to several types of stress. ns = not significant.

**a. Oxidative stress**				
**PDT-treated vs. non-treated HT29 tumor cells at 24 h post-PDT**				
**Gene**	**Adhered cells**		**Detached cells**	
	**FR**	***p*** **value**	**FR**	***p*** **value**
*HMOX1*	8.87	<0.05	7.91	<0.01
*FTH1*	2.38	<0.05	5.41	<0.01
*GCLC*	3.36	<0.05	2.74	<0.01
*GCLM*	2.85	<0.05	3.96	<0.001
*GSR*	2.01	<0.01	2.85	<0.001
*SQSTM1*	4.98	<0.001	7.49	<0.001
*PRDX1*	ns	ns	4.18	<0.001
*NQO1*	ns	ns	3.02	<0.01
*TXN*	ns	ns	3.29	0,0.05
*GSTP1*	ns	ns	2.41	<0.01
*TXNRD1*	4.16	<0.001	ns	ns
**b. Hypoxia signaling**				
**PDT-treated vs. non-treated HT29 tumor cells at 24 h post-PDT**				
**Gene**	**Adhered cells**		**Detached cells**	
	**FR**	***p*** **value**	**FR**	***p*** **value**
*HMOX1*	8.87	<0.05	7.91	<0.01
*SERPINE1*	9.78	<0.001	10.74	<0.001
*ADM*	4.60	<0.01	14.27	<0.001
*ARNT*	ns	ns	2.36	<0.05
*VEGFA*	1.81	ns	2.11	≤0.001
*BNIP3L*	2.02	ns	2.20	<0.05
**c. Cell death**				
**PDT-treated vs. non-treated HT29 tumor cells at 24 h post-PDT**				
**Gene**	**Adhered cells**		**Detached cells**	
	**FR**	***p*** **value**	**FR**	***p*** **value**
***Apoptosis***				
*TNFRSF10B (DR5/TRAILR2)*	3.09	<0.001	2.86	<0.001
*BID*	1.75	ns	3.96	<0.05
*BNIP3L*	2.02	ns	2.20	<0.05
*BBC3*	4.19	<0.05	5.30	ns
***Necrosis***				
*RIPK1*	1.78	<0.01	ns	ns
***Autophagy***				
*ATG12*	1.85	<0.05	3.29	<0.01
*SQSTM1*	4.98	<0.001	7.49	<0.001
**d. DNA damage responses**				
**PDT-treated vs. non-treated HT29 tumor cells at 24 h post-PDT**				
**Gene**	**Adhered cells**		**Detached cells**	
	**FR**	***p*** **value**	**FR**	***p*** **value**
***Cell cycle arrest***				
*CDKN1A*	2.74	<0.05	5.29	<0.01
*GADD45A*	3.01	<0.001	2.35	<0.001
*DDIT3 (GADD153)*	4.77	<0.01	12.86	<0.01
*GADD45G*	3.01	ns	14.77	<0.05
*CHEK2*	ns	ns	1.71	<0.05
*HUS1*	1.62	<0.01	ns	ns
***Other DNA damage responses***				
*DDB2*	1.51	<0.05	2.63	<0.01
*XPC*	2.20	<0.05	1.67	ns
**e. Unfolded protein response**				
**PDT-treated vs. non-treated HT29 tumor cells at 24 h post-PDT**				
**Gene**	**Adhered cells**		**Detached cells**	
	**FR**	***p*** **value**	**FR**	***p*** **value**
*HSP90AA1*	16.52	<0.05	9.26	<0.01
*DDIT3 (CHOP)*	4.77	<0.01	12.86	<0.01
*BBC3*	4.19	<0.05	5.30	ns
*BID*	1.75	ns	3.96	<0.05
*HSPA4 (HSP70)*	2.96	ns	2.95	<0.05
*HSPA5 (GRP78)*	−2.27	<0.01	−2.42	<0.001
*DNAJC3*	−1.59	<0.05	−4.07	<0.001
*HSP90B1*	−1.77	ns	−3.59	<0.001
*CALR*	−1.89	<0.001	ns	ns
**f. Inflammation**				
**PDT-treated vs. non-treated HT29 tumor cells at 24 h post-PDT**				
**Gene**	**Adhered cells**		**Detached cells**	
	**FR**	***p*** **value**	**FR**	***p*** **value**
*CXCL8*	8.09	≤0.01	6.46	<0.01
*IL1B*	3.28	<0.001	2.15	ns
*IL1A*	ns	ns	−2.90	≤0.01

## Data Availability

The data presented in this study are presented in the paper, and raw data are available on request from the corresponding author.

## References

[1] Agostinis P., Berg K., Cengel K.A., Foster T.H., Girotti A.W., Gollnick S.O., Hahn S.M., Hamblin M.R., Juzeniene A., Kessel D. (2011). Photodynamic therapy of cancer: An update. CA. Cancer J. Clin..

[2] Falk-Mahapatra R., Gollnick S.O. (2020). Photodynamic Therapy and Immunity: An Update. Photochem. Photobiol..

[3] Manda G., Hinescu M.E., Neagoe I.V., Ferreira L.F.V., Boscencu R., Vasos P., Basaga S.H., Cuadrado A. (2018). Emerging Therapeutic Targets in Oncologic Photodynamic Therapy. Curr. Pharm. Des..

[4] Li X.-Y., Tan L.-C., Dong L.-W., Zhang W.-Q., Shen X.-X., Lu X., Zheng H., Lu Y.-G. (2020). Susceptibility and Resistance Mechanisms During Photodynamic Therapy of Melanoma. Front. Oncol..

[5] Donohoe C., Senge M.O., Arnaut L.G., Gomes-da-Silva L.C. (2019). Cell death in photodynamic therapy: From oxidative stress to anti-tumor immunity. Biochim. Biophys. Acta Rev. Cancer.

[6] Garg A.D., Krysko D.V., Vandenabeele P., Agostinis P. (2011). DAMPs and PDT-mediated photo-oxidative stress: Exploring the unknown. Photochem. Photobiol. Sci..

[7] Bigot E., Bataille R., Patrice T. (2012). Increased singlet oxygen-induced secondary ROS production in the serum of cancer patients. J. Photochem. Photobiol. B.

[8] Boscencu R., Manda G., Radulea N., Socoteanu R.P., Ceafalan L.C., Neagoe I.V., Ferreira Machado I., Basaga S.H., Vieira Ferreira L.F. (2017). Studies on the Synthesis, Photophysical and Biological Evaluation of Some Unsymmetrical Meso-Tetrasubstituted Phenyl Porphyrins. Molecules.

[9] Chan F.K.-M., Moriwaki K., De Rosa M.J. (2013). Detection of necrosis by release of lactate dehydrogenase activity. Methods Mol. Biol..

[10] Ros U., Peña-Blanco A., Hänggi K., Kunzendorf U., Krautwald S., Wong W.W.-L., García-Sáez A.J. (2017). Necroptosis Execution Is Mediated by Plasma Membrane Nanopores Independent of Calcium. Cell Rep..

[11] Castano A.P., Demidova T.N., Hamblin M.R. (2004). Mechanisms in photodynamic therapy: Part one-photosensitizers, photochemistry and cellular localization. Photodiagn. Photodyn. Ther..

[12] Looft A., Pfitzner M., Preuß A., Röder B. (2018). In vivo singlet molecular oxygen measurements: Sensitive to changes in oxygen saturation during PDT. Photodiagn. Photodyn. Ther..

[13] Shen Y., Li X., Zhao B., Xue Y., Wang S., Chen X., Yang J., Lv H., Shang P. (2018). Iron metabolism gene expression and prognostic features of hepatocellular carcinoma. J. Cell. Biochem..

[14] Bachhawat A.K., Yadav S. (2018). The glutathione cycle: Glutathione metabolism beyond the γ-glutamyl cycle. IUBMB Life.

[15] Mohammadi F., Soltani A., Ghahremanloo A., Javid H., Hashemy S.I. (2019). The thioredoxin system and cancer therapy: A review. Cancer Chemother. Pharmacol..

[16] Li H.-X., Sun X.-Y., Yang S.-M., Wang Q., Wang Z.-Y. (2018). Peroxiredoxin 1 promoted tumor metastasis and angiogenesis in colorectal cancer. Pathol. Res. Pract..

[17] Ji L., Wei Y., Jiang T., Wang S. (2014). Correlation of Nrf2, NQO1, MRP1, cmyc and p53 in colorectal cancer and their relationships to clinicopathologic features and survival. Int. J. Clin. Exp. Pathol..

[18] Beaver S.K., Mesa-Torres N., Pey A.L., Timson D.J. (2019). NQO1: A target for the treatment of cancer and neurological diseases, and a model to understand loss of function disease mechanisms. Biochim. Biophys. Acta Proteins Proteom..

[19] Kosumi K., Masugi Y., Yang J., Qian Z.R., Kim S.A., Li W., Shi Y., da Silva A., Hamada T., Liu L. (2017). Tumor SQSTM1 (p62) expression and T cells in colorectal cancer. Oncoimmunology.

[20] Zhang J., Yang S., Xu B., Wang T., Zheng Y., Liu F., Ren F., Jiang J., Shi H., Zou B. (2019). p62 functions as an oncogene in colorectal cancer through inhibiting apoptosis and promoting cell proliferation by interacting with the vitamin D receptor. Cell Prolif..

[21] Li S., Wei X., He J., Tian X., Yuan S., Sun L. (2018). Plasminogen activator inhibitor-1 in cancer research. Biomed. Pharmacother..

[22] Wang L., Gala M., Yamamoto M., Pino M.S., Kikuchi H., Shue D.S., Shirasawa S., Austin T.R., Lynch M.P., Rueda B.R. (2014). Adrenomedullin is a therapeutic target in colorectal cancer. Int. J. Cancer.

[23] Tsai Y.-P., Wu K.-J. (2014). Epigenetic regulation of hypoxia-responsive gene expression: Focusing on chromatin and DNA modifications. Int. J. Cancer.

[24] Miyazaki S., Kikuchi H., Iino I., Uehara T., Setoguchi T., Fujita T., Hiramatsu Y., Ohta M., Kamiya K., Kitagawa K. (2014). Anti-VEGF antibody therapy induces tumor hypoxia and stanniocalcin 2 expression and potentiates growth of human colon cancer xenografts. Int. J. Cancer.

[25] Fei P., Wang W., Kim S., Wang S., Burns T.F., Sax J.K., Buzzai M., Dicker D.T., McKenna W.G., Bernhard E.J. (2004). Bnip3L is induced by p53 under hypoxia, and its knockdown promotes tumor growth. Cancer Cell.

[26] Chau L.-Y. (2015). Heme oxygenase-1: Emerging target of cancer therapy. J. Biomed. Sci..

[27] Alam J., Cook J.L. (2007). How many transcription factors does it take to turn on the heme oxygenase-1 gene?. Am. J. Respir. Cell Mol. Biol..

[28] Babinčák M., Jendželovský R., Košuth J., Majerník M., Vargová J., Mikulášek K., Zdráhal Z., Fedoročko P. (2021). Death Receptor 5 (TNFRSF10B) Is Upregulated and TRAIL Resistance Is Reversed in Hypoxia and Normoxia in Colorectal Cancer Cell Lines after Treatment with Skyrin, the Active Metabolite of Hypericum spp.. Cancers.

[29] Gahl R.F., Dwivedi P., Tjandra N. (2016). Bcl-2 proteins bid and bax form a network to permeabilize the mitochondria at the onset of apoptosis. Cell Death Dis..

[30] Yu J., Zhang L., Hwang P.M., Kinzler K.W., Vogelstein B. (2001). PUMA induces the rapid apoptosis of colorectal cancer cells. Mol. Cell.

[31] Wang L., Chang X., Feng J., Yu J., Chen G. (2019). TRADD Mediates RIPK1-Independent Necroptosis Induced by Tumor Necrosis Factor. Front. Cell Dev. Biol..

[32] Hu J.L., He G.Y., Lan X.L., Zeng Z.C., Guan J., Ding Y., Qian X.L., Liao W.T., Ding Y.Q., Liang L. (2018). Inhibition of ATG12-mediated autophagy by miR-214 enhances radiosensitivity in colorectal cancer. Oncogenesis.

[33] Bjørkøy G., Lamark T., Pankiv S., Øvervatn A., Brech A., Johansen T. (2009). Monitoring autophagic degradation of p62/SQSTM1. Methods Enzymol..

[34] Cazzalini O., Scovassi A.I., Savio M., Stivala L.A., Prosperi E. (2010). Multiple roles of the cell cycle inhibitor p21(CDKN1A) in the DNA damage response. Mutat. Res..

[35] Salvador J.M., Brown-Clay J.D., Fornace A.J. (2013). Gadd45 in stress signaling, cell cycle control, and apoptosis. Adv. Exp. Med. Biol..

[36] Xiang H., Geng X., Ge W., Li H. (2011). Meta-analysis of CHEK2 1100delC variant and colorectal cancer susceptibility. Eur. J. Cancer.

[37] Zhou Z.-Q., Zhao J.-J., Chen C.-L., Liu Y., Zeng J.-X., Wu Z.-R., Tang Y., Zhu Q., Weng D.-S., Xia J.-C. (2019). HUS1 checkpoint clamp component (HUS1) is a potential tumor suppressor in primary hepatocellular carcinoma. Mol. Carcinog..

[38] Jauhiainen A., Thomsen C., Strömbom L., Grundevik P., Andersson C., Danielsson A., Andersson M.K., Nerman O., Rörkvist L., Ståhlberg A. (2012). Distinct cytoplasmic and nuclear functions of the stress induced protein DDIT3/CHOP/GADD153. PLoS ONE.

[39] Bagchi S., Raychaudhuri P. (2010). Damaged-DNA Binding Protein-2 Drives Apoptosis Following DNA Damage. Cell Div..

[40] Nemzow L., Lubin A., Zhang L., Gong F. (2015). XPC: Going where no DNA damage sensor has gone before. DNA Repair.

[41] Rashid H.-O., Yadav R.K., Kim H.-R., Chae H.-J. (2015). ER stress: Autophagy induction, inhibition and selection. Autophagy.

[42] Reimertz C., Kögel D., Rami A., Chittenden T., Prehn J.H.M. (2003). Gene expression during ER stress-induced apoptosis in neurons: Induction of the BH3-only protein Bbc3/PUMA and activation of the mitochondrial apoptosis pathway. J. Cell Biol..

[43] Ma Y., Hendershot L.M. (2004). ER chaperone functions during normal and stress conditions. J. Chem. Neuroanat..

[44] Theodoraki M.A., Caplan A.J. (2012). Quality control and fate determination of Hsp90 client proteins. Biochim. Biophys. Acta.

[45] Ibrahim I.M., Abdelmalek D.H., Elfiky A.A. (2019). GRP78: A cell’s response to stress. Life Sci..

[46] Jackson S.E. (2013). Hsp90: Structure and function. Top. Curr. Chem..

[47] Michalak M., Groenendyk J., Szabo E., Gold L.I., Opas M. (2009). Calreticulin, a multi-process calcium-buffering chaperone of the endoplasmic reticulum. Biochem. J..

[48] Van Huizen R., Martindale J.L., Gorospe M., Holbrook N.J. (2003). P58IPK, a novel endoplasmic reticulum stress-inducible protein and potential negative regulator of eIF2alpha signaling. J. Biol. Chem..

[49] Feng R., Ye J., Zhou C., Qi L., Fu Z., Yan B., Liang Z., Li R., Zhai W. (2015). Calreticulin down-regulation inhibits the cell growth, invasion and cell cycle progression of human hepatocellular carcinoma cells. Diagn. Pathol..

[50] Cohen I., Idan C., Rider P., Peleg R., Vornov E., Elena V., Tomas M., Martin T., Tudor C., Cicerone T. (2015). IL-1α is a DNA damage sensor linking genotoxic stress signaling to sterile inflammation and innate immunity. Sci. Rep..

[51] Nair P., Lu M., Petersen S., Ashkenazi A. (2014). Apoptosis Initiation Through the Cell-Extrinsic Pathway. Methods Enzymol..

[52] Oliver P.G., LoBuglio A.F., Zinn K.R., Kim H., Nan L., Zhou T., Wang W., Buchsbaum D.J. (2008). Treatment of human colon cancer xenografts with TRA-8 anti-death receptor 5 antibody alone or in combination with CPT-11. Clin. Cancer Res..

[53] She T., Shi Q., Li Z., Feng Y., Yang H., Tao Z., Li H., Chen J., Wang S., Liang Y. (2021). Combination of long-acting TRAIL and tumor cell-targeted photodynamic therapy as a novel strategy to overcome chemotherapeutic multidrug resistance and TRAIL resistance of colorectal cancer. Theranostics.

[54] Billen L.P., Shamas-Din A., Andrews D.W. (2008). Bid: A Bax-like BH3 protein. Oncogene.

[55] Han J., Flemington C., Houghton A.B., Gu Z., Zambetti G.P., Lutz R.J., Zhu L., Chittenden T. (2001). Expression of bbc3, a pro-apoptotic BH3-only gene, is regulated by diverse cell death and survival signals. Proc. Natl. Acad. Sci. USA.

[56] Sun Y., Zhao D., Wang G., Wang Y., Cao L., Sun J., Jiang Q., He Z. (2020). Recent progress of hypoxia-modulated multifunctional nanomedicines to enhance photodynamic therapy: Opportunities, challenges, and future development. Acta Pharm. Sin. B.

[57] Gong Y., Fan Z., Luo G., Yang C., Huang Q., Fan K., Cheng H., Jin K., Ni Q., Yu X. (2019). The role of necroptosis in cancer biology and therapy. Mol. Cancer.

[58] Newton K. (2015). RIPK1 and RIPK3: Critical regulators of inflammation and cell death. Trends Cell Biol..

[59] Martins W.K., Belotto R., Silva M.N., Grasso D., Suriani M.D., Lavor T.S., Itri R., Baptista M.S., Tsubone T.M. (2020). Autophagy Regulation and Photodynamic Therapy: Insights to Improve Outcomes of Cancer Treatment. Front. Oncol..

[60] Huang R.-X., Zhou P.-K. (2020). DNA damage response signaling pathways and targets for radiotherapy sensitization in cancer. Signal Transduct. Target. Ther..

[61] Wang J.Y.J. (2019). Cell Death Response to DNA Damage. Yale J. Biol. Med..

[62] Kumar N., Raja S., Van Houten B. (2020). The involvement of nucleotide excision repair proteins in the removal of oxidative DNA damage. Nucleic Acids Res..

[63] Stoyanova T., Roy N., Kopanja D., Raychaudhuri P., Bagchi S. (2009). DDB2 (damaged-DNA binding protein 2) in nucleotide excision repair and DNA damage response. Cell Cycle.

[64] Barakat B.M., Wang Q.-E., Han C., Milum K., Yin D.-T., Zhao Q., Wani G., Arafa E.-S.A., El-Mahdy M.A., Wani A.A. (2010). Overexpression of DDB2 enhances the sensitivity of human ovarian cancer cells to cisplatin by augmenting cellular apoptosis. Int. J. Cancer.

[65] Roy N., Bagchi S., Raychaudhuri P. (2012). Damaged DNA binding protein 2 in reactive oxygen species (ROS) regulation and premature senescence. Int. J. Mol. Sci..

[66] Minig V., Kattan Z., van Beeumen J., Brunner E., Becuwe P. (2009). Identification of DDB2 protein as a transcriptional regulator of constitutive SOD2 gene expression in human breast cancer cells. J. Biol. Chem..

[67] Tomioka Y., Kushibiki T., Awazu K. (2010). Evaluation of oxygen consumption of culture medium and in vitro photodynamic effect of talaporfin sodium in lung tumor cells. Photomed. Laser Surg..

[68] Hernansanz-Agustín P., Izquierdo-Álvarez A., Sánchez-Gómez F.J., Ramos E., Villa-Piña T., Lamas S., Bogdanova A., Martínez-Ruiz A. (2014). Acute hypoxia produces a superoxide burst in cells. Free Radic. Biol. Med..

[69] Jung S.-N., Yang W.K., Kim J., Kim H.S., Kim E.J., Yun H., Park H., Kim S.S., Choe W., Kang I. (2008). Reactive oxygen species stabilize hypoxia-inducible factor-1 alpha protein and stimulate transcriptional activity via AMP-activated protein kinase in DU145 human prostate cancer cells. Carcinogenesis.

[70] Muz B., de la Puente P., Azab F., Azab A.K. (2015). The role of hypoxia in cancer progression, angiogenesis, metastasis, and resistance to therapy. Hypoxia.

[71] Lin Y., Jiang M., Chen W., Zhao T., Wei Y. (2019). Cancer and ER stress: Mutual crosstalk between autophagy, oxidative stress and inflammatory response. Biomed. Pharmacother..

[72] Moserova I., Kralova J. (2012). Role of ER stress response in photodynamic therapy: ROS generated in different subcellular compartments trigger diverse cell death pathways. PLoS ONE.

[73] Codogno P., Meijer A.J. (2005). Autophagy and signaling: Their role in cell survival and cell death. Cell Death Differ..

[74] Sano R., Reed J.C. (2013). ER stress-induced cell death mechanisms. Biochim. Biophys. Acta.

[75] Lei Y., Wang S., Ren B., Wang J., Chen J., Lu J., Zhan S., Fu Y., Huang L., Tan J. (2017). CHOP favors endoplasmic reticulum stress-induced apoptosis in hepatocellular carcinoma cells via inhibition of autophagy. PLoS ONE.

[76] Riggs S., Alario A.J., McHorney C. (1990). Health risk behaviors and attempted suicide in adolescents who report prior maltreatment. J. Pediatr..

[77] Pereira E.J., Burns J.S., Lee C.Y., Marohl T., Calderon D., Wang L., Atkins K.A., Wang C.-C., Janes K.A. (2020). Sporadic activation of an oxidative stress-dependent NRF2-p53 signaling network in breast epithelial spheroids and premalignancies. Sci. Signal..

[78] Chen W., Sun Z., Wang X.-J., Jiang T., Huang Z., Fang D., Zhang D.D. (2009). Direct interaction between Nrf2 and p21(Cip1/WAF1) upregulates the Nrf2-mediated antioxidant response. Mol. Cell.

[79] Choi B., Ryoo I., Kang H.C., Kwak M.-K. (2014). The sensitivity of cancer cells to pheophorbide a-based photodynamic therapy is enhanced by Nrf2 silencing. PLoS ONE.

[80] Robledinos-Antón N., Fernández-Ginés R., Manda G., Cuadrado A. (2019). Activators and Inhibitors of NRF2: A Review of Their Potential for Clinical Development. Oxid. Med. Cell. Longev..

[81] Zawacka-Pankau J., Krachulec J., Grulkowski I., Bielawski K.P., Selivanova G. (2008). The p53-mediated cytotoxicity of photodynamic therapy of cancer: Recent advances. Toxicol. Appl. Pharmacol..

[82] Kruiswijk F., Labuschagne C.F., Vousden K.H. (2015). p53 in survival, death and metabolic health: A lifeguard with a licence to kill. Nat. Rev. Mol. Cell Biol..

[83] Eriksson S.E., Ceder S., Bykov V.J.N., Wiman K.G. (2019). p53 as a hub in cellular redox regulation and therapeutic target in cancer. J. Mol. Cell Biol..

[84] Pezzuto A., Carico E. (2018). Role of HIF-1 in Cancer Progression: Novel Insights. A Review. Curr. Mol. Med..

[85] Lamberti M.J., Pansa M.F., Vera R.E., Fernández-Zapico M.E., Rumie Vittar N.B., Rivarola V.A. (2017). Transcriptional activation of HIF-1 by a ROS-ERK axis underlies the resistance to photodynamic therapy. PLoS ONE.

[86] Li T., Su L., Lei Y., Liu X., Zhang Y., Liu X. (2015). DDIT3 and KAT2A Proteins Regulate TNFRSF10A and TNFRSF10B Expression in Endoplasmic Reticulum Stress-mediated Apoptosis in Human Lung Cancer Cells. J. Biol. Chem..

[87] Yamaguchi H., Wang H.-G. (2004). CHOP is involved in endoplasmic reticulum stress-induced apoptosis by enhancing DR5 expression in human carcinoma cells. J. Biol. Chem..

[88] Sullivan G.P., O’Connor H., Henry C.M., Davidovich P., Clancy D.M., Albert M.L., Cullen S.P., Martin S.J. (2020). TRAIL Receptors Serve as Stress-Associated Molecular Patterns to Promote ER-Stress-Induced Inflammation. Dev. Cell.

[89] Greten F.R., Grivennikov S.I. (2019). Inflammation and Cancer: Triggers, Mechanisms, and Consequences. Immunity.

[90] Jundi K., Greene C.M. (2015). Transcription of Interleukin-8: How Altered Regulation Can Affect Cystic Fibrosis Lung Disease. Biomolecules.

[91] Russo R.C., Garcia C.C., Teixeira M.M., Amaral F.A. (2014). The CXCL8/IL-8 chemokine family and its receptors in inflammatory diseases. Expert Rev. Clin. Immunol..

[92] Galdiero M.R., Bianchi P., Grizzi F., Di Caro G., Basso G., Ponzetta A., Bonavita E., Barbagallo M., Tartari S., Polentarutti N. (2016). Occurrence and significance of tumor-associated neutrophils in patients with colorectal cancer. Int. J. Cancer.

[93] Berry R.S., Xiong M.-J., Greenbaum A., Mortaji P., Nofchissey R.A., Schultz F., Martinez C., Luo L., Morris K.T., Hanson J.A. (2017). High levels of tumor-associated neutrophils are associated with improved overall survival in patients with stage II colorectal cancer. PLoS ONE.

[94] Arelaki S., Arampatzioglou A., Kambas K., Papagoras C., Miltiades P., Angelidou I., Mitsios A., Kotsianidis I., Skendros P., Sivridis E. (2016). Gradient Infiltration of Neutrophil Extracellular Traps in Colon Cancer and Evidence for Their Involvement in Tumour Growth. PLoS ONE.

[95] Bie Y., Ge W., Yang Z., Cheng X., Zhao Z., Li S., Wang W., Wang Y., Zhao X., Yin Z. (2019). The Crucial Role of CXCL8 and Its Receptors in Colorectal Liver Metastasis. Dis. Markers.

[96] Samuel T., Fadlalla K., Gales D.N., Putcha B.D.K., Manne U. (2014). Variable NF-κB pathway responses in colon cancer cells treated with chemotherapeutic drugs. BMC Cancer.

[97] Ferreira L.F.V., Machado I.F., Gama A., Socoteanu R.P., Boscencu R., Manda G., Calhelha R.C., Ferreira I.C.F.R. (2020). Photochemical /Photocytotoxicity Studies of New Tetrapyrrolic Structures as Potential Candidates for Cancer Theranostics. Curr. Drug Discov. Technol..

[98] Xie F., Xiao P., Chen D., Xu L., Zhang B. (2012). miRDeepFinder: A miRNA analysis tool for deep sequencing of plant small RNAs. Plant Mol. Biol..

